# Antenatal care visit attendance, intermittent preventive treatment during pregnancy (IPTp) and malaria parasitaemia at delivery

**DOI:** 10.1186/1475-2875-13-162

**Published:** 2014-04-30

**Authors:** Judith K Anchang-Kimbi, Eric A Achidi, Tobias O Apinjoh, Regina N Mugri, Hanesh Fru Chi, Rolland B Tata, Blaise Nkegoum, Joseph-Marie N Mendimi, Eva Sverremark-Ekström, Marita Troye-Blomberg

**Affiliations:** 1 Department of Zoology and Animal Physiology, University of Buea, Buea 63, Cameroon; 2 Department of Biochemistry and Molecular Biology, University of Buea, Buea 63, Cameroon; 3 Department of Microbiology and Parasitology, University of Buea, Buea 63, Cameroon; 4 Department of Anatomy and Pathology, University of Yaoundé Teaching Hospital, Yaoundé 63, Cameroon; 5 Department of Molecular Bioscience, Wenner-Gren Institute, Stockholm University, SE- 10691 Stockholm, Sweden

**Keywords:** Antenatal care visit attendance, IPTp, Malaria parasite infection

## Abstract

**Background:**

The determinants and barriers for delivery and uptake of IPTp vary with different regions in sub-Saharan Africa. This study evaluated the determinants of ANC clinic attendance and IPTp-SP uptake among parturient women from Mount Cameroon Area and hypothesized that time of first ANC clinic attendance could influence uptake of IPTp-SP/dosage and consequently malaria parasite infection status at delivery.

**Methods:**

Two cross sectional surveys were carried out at the Government Medical Centre in the Mutengene Health Area, Mt Cameroon Area from March to October 2007 and June 2008 to April 2009. Consented parturient women were consecutively enrolled in both surveys. In 2007, socio-demographic data, ANC clinic attendance, gestational age, fever history and reported use/dosage of IPTp-SP were documented using a structured questionnaire. In the second survey only IPT-SP usage/dosage was recorded. Malaria parasitaemia at delivery was determined by blood smear microscopy and placental histology.

**Results and discussion:**

In 2007, among the 287 women interviewed, 2.2%, 59.7%, and 38.1% enrolled in the first, second and third trimester respectively. About 90% of women received at least one dose SP but only 53% received the two doses in 2007 and by 2009 IPTp-two doses coverage increased to 64%. Early clinic attendance was associated (P = 0.016) with fever history while being unmarried (OR = 2.2; 95% CI: 1.3-3.8) was significantly associated with fewer clinic visits (<4visits). Women who received one SP dose (OR = 3.7; 95% CI: 2.0-6.8) were more likely not to have attended ≥ 4visits. A higher proportion (P < 0.001) of women with first visit during the third trimester received only one dose, meanwhile, those who had an early first ANC attendance were more likely (OR = 0.4; 95% CI = 0.2 - 0.7) to receive two or more doses. Microscopic parasitaemia at delivery was frequent (P = 0.007) among women who enrolled in the third trimester and had received only one SP dose than in those with two doses.

**Conclusion:**

In the study area, late first ANC clinic enrolment and fewer clinic visits may prevent the uptake of two SP doses and education on early and regular ANC clinic visits can increase IPTp coverage.

## Background

Antenatal care is an important component of maternal and child health care to monitor the health of the mother, anticipate difficulties and complications of labour, ensure the birth of a healthy baby and to help the mother rear the child [1]. Infrequent, late or non-attendance at antenatal care clinics has been associated with adverse maternal outcome [2]. Malaria in pregnancy remains a major public health problem in endemic areas of the African region, where approximately 25–30 million of pregnant women are at risk of *Plasmodium falciparum* infection and its adverse outcomes during their pregnancy [3,4]. To reduce malaria burden in pregnancy, the World Health Organization (WHO) currently recommends a package of interventions in areas with stable (high) transmission of *P. falciparum*[4]. These include the use of insecticide-treated nets (ITNs), intermittent preventive treatment (IPT) and effective case management of malaria illness and anaemia. Currently, sulphadoxine-pyrimethamine (SP) is the most effective drug for IPT despite the increasing resistance of SP to *P. falciparum*[5,6]. Intermittent preventive therapy with SP for malaria in pregnancy (IPTp-SP) in areas of high or seasonal transmission have been shown to lower placental infection rates, increase both maternal haemoglobin levels and the infants’ birth weight [7,8].

A high proportion of pregnant women in Africa attend ANC clinic at least once during pregnancy thus the antenatal clinic provides a good opportunity for delivering interventions to control malaria in pregnancy in endemic regions. Studies conducted in Malawi and Kenya showed that the maximum benefit of IPT can be gained by receiving two doses or more (in HIV infected pregnant women) doses of SP. More so, majority of women who received a minimum of two doses of SP do attend four or more ANC visits [9]. Despite a high ANC clinic attendance in most countries in Sub-Africa, reported coverage level of IPTp-SP and ITNs remains below the reversed Abuja Declaration target of 80% [10].

The determinants and barriers for delivery and uptake of IPT and ITN vary with different regions [11]. Meta-analysis of factors affecting the delivery, access, and use of interventions to prevent malaria in pregnancy in sub-Saharan Africa identified education, knowledge and perception about malaria [12,13], socio-economic status [14-17], number and timing of antenatal clinic visits [12,17], number of pregnancies, healthcare system issues [13,17] as factors that influence intervention coverage as well as access to ANC services. While most reports on ANC clinic attendance and IPTp/SP coverage are from studies carried out in West and East Africa, reports from Central Africa are limited [17].

In 2007, about 90% of women in Mount Cameroon Area attending ANC clinic received IPTp-SP at least once during pregnancy, but only 52% of these women had the recommended two or more doses [18]. To identify facilitating factors for scaling up IPTp-SP coverage in the study area, this study evaluated the determinants of ANC clinic attendance and IPTp-SP uptake among parturient women who seek delivery services at the Mutengene Medical Centre. The study hypothesized that time of first ANC clinic attendance could influence uptake of IPTp-SP/dosage and consequently malaria parasite infection status among pregnant women at delivery.

## Methods

### Study area and population

This study consists of two cross sectional surveys carried out at the Government Medical Centre in the Mutengene Health Area, Mt Cameroon Area, Southwest Region from March to October 2007 and June 2008 to April 2009. The Mutengene Medical Centre is the only government owned institution that offers antenatal care, preventive and curative services at subsidized costs in the health area. The characteristics of the study area have been described elsewhere [18]. In brief, malaria transmission is perennial, with some seasonality. Pregnant women in this semi-urban area of Cameroon are frequently (62%) exposed to *P. falciparum* infection during pregnancy with placental malaria parasitaemia rate of 34% at delivery. History of fever attack is common (44%) in women during pregnancy [18]. Previous studies show that anaemia is still a major health problem in this area [18-20]. Consecutive pregnant women reporting for delivery at the Mutengene maternity and who consented to participate in the study were enrolled.

### Administration of questionnaires

During the first survey*,* a total of 287 parturient women were interviewed. A structured questionnaire was administered to women to document socio-demographic data including age, residence, marital status and educational level. Antenatal data were obtained from either the antenatal card or the register of the centre with assistance of a midwife and included: parity, gestational age at first ANC, number of ANC visits made. Gestational age was determined after the physical examination by the midwife who used a gestational calendar or uterine growth when the date of last menses was not known. Fever history, use of IPTpSP (dosage and dates of drug administration) was recorded from ANC cards, patient’s medical record book and health centre maternal care register. During the second survey, only IPTp-SP usage/dosage was recorded for 292 pregnant women at ≥ 36 weeks of gestation.

### Sample collection and processing

Maternal peripheral venous blood (2 ml) was collected within 24 hours after delivery for malaria parasite detection. Immediately following delivery, the placenta was obtained and a small piece of placental tissue (0.5 cm^3^) excised from the centre of the placenta to prepare impression smears. A larger (4 cm^3^) biopsy specimen of placental tissue was fixed in 10% neutral buffered formalin for histopathological assessment as described elsewhere [18].

### Parasitological examination

Peripheral blood was used to prepare thick blood smears and together with placental impression smears stained with Giemsa (Sigma) and examined by light microscopy. Placental tissue sections were processed, stained with haematoxylin and eosin and examined. Placental malaria parasitisation was determined as reported elsewhere [18].

### Definitions and data analysis

All data were entered and analysed using SPSS version 19. Age and parity was categorized as follows: age (≤20, 21–25, >25) years; parity (primiparae, secundiparae and multiparae (≥3 pregnancies). The Complete ANC attendance was considered to be at least four ANC visits during pregnancy. A full course IPTp/SP was defined as at least a complete two-dose course of SP administered during the pregnancy. Malaria infection was defined as the presence of parasites detected by peripheral blood, placental blood microscopy and placental histologic examination further classified as microscopic and submicroscopic infection.

Differences between groups were assessed using chi-square or Fisher’s exact tests for proportions and student *t*-test for means. The factors that influence ANC clinic attendance and IPTp-SP uptake were assessed using odds ratio and the 95% confidence interval (OR (95% CI) determined in univariate analysis and logistic regression in multivariate analysis. Correlations were assessed using Pearson correlation test. A p-value of less than 0.05 was considered significant. The study received ethical clearance from the Delegation of Public Health Ethics Review Board, Buea, Southwest Region.

## Results

### Characteristics of study participants

The characteristics of parturient women who participated in the study in 2007 are shown in (Table 1). Their ages range from 14–40 years with a median age and parity of 23(20–27) years and 2(1–3) births respectively. There was strong positive correlation (r = 0.796; P < 0.001) between age and parity of all mothers, where primiparous mothers (19.6 ± 2.7 years) were significantly younger (p < 0.001) compared to multiparous mothers (25.8 ± 4.7 years). The study population was predominantly made up of married women (75.1%); and 62.3% of the women were educated to primary level and only 4.5% and 6.4% had tertiary level and no formal education respectively. It is noted that 59.2% (125/211) of the married women were housewives and engaged in no occupation while 52.2% (35/67) of the unmarried mothers were students or applicants. At delivery, the median gestational age of 39 (38–40) weeks and neonatal birth weight of 3.20 (3.00-3.60) kg respectively were recorded.

**Table 1:** Socio-demographic characteristics of participants (N = 287)

**Factor**	**% (n)**
**Age (years)**	
≤20	30.3 (86)
21-25	35.9 (102)
>25	33.8 (96)
**Parity**	
Primiparous	30.7 (88)
Secundiparous	28.9 (83)
Multiparous	40.4 (116)
**Educational level**	
None	6.4 (17)
Primary	62.3 (165)
Secondary	26.8 (71)
Tertiary	4.5 (12)
**Marital status**	
Married	75.1 (214)
Unmarried	24.9 (71)
**Occupation**	
Housewife	45.9 (128)
Student	16.5 (46)
Business	30.8 (86)
Farming	5.4 (15)
Profession	1.4 (4)

### Antenatal care attendance

Of the 287 participating women, 96.5% reported attending ANC at least once during their last recent pregnancy. Antenatal clinic visits varied from 1–12 with a median of 5 visits. Among ANC attendees, majority (60%) made their first visit in the second trimester (Table 2). Approximately 91% of the women made more than one visit and only 69.3% of the ANC attendees had complete attendance (considered to be at least four ANC visits during pregnancy) (Table 2). Women who had a complete ANC attendance were significantly older (P = 0.038) when compared with those who did fewer ANC visits. Also married women frequently made four or more visits than unmarried women. Thus younger age (≤20 years) (OR = 2.0; 95% CI: 1.2-3.4) and being unmarried (OR = 2.2; 95% CI: 1.3-3.8) were significant risk factors associated with fewer clinic visits (<4visits). Fever history was associated with more ANC clinic visits (OR = 0.51, 95% CI 0.30 - 0.89) (Table 3) as women with fever history (22.13 ± 4.73 weeks) reported earlier (t = − 4.790; P < 0.001) for first ANC clinic visit when compared with those with no fever history (25.00 ± 4.80 weeks). In multivariate analysis, marital status (unmarried) (p = 0.006) and fever history (P = 0.009) remained significant independent factors associated with ANC visit attendance (Table 3).

**Table 2:** Antenatal care visit attendance and IPTp-SP usage of parturient women (N = 287)

**Characteristics**	**% (n)**	
**ANC visit at least once**	96.5 (277)	
**ANC visit at least twice**	94.2 (261)	
**ANC visit at least thrice**	84.1 (233)	
**≥ 4 ANC visits**	71.8 (199)	
**Median No. of ANC visits (quartiles)**	5 (3–6)	
**Non-ANC attendance**	3.5 (10)	
**First ANC visit; Median GA (range)**		
1^st^ trimester	2.2 (06)	12 (8–12)
2^nd^ trimester	59.7 (160)	21 (14–25)
3^rd^ trimester	38.1 (102)	29 (26–37)
**IPTp-SP usage**	90.5 (258/285)	
**SP dosage**		
Two or more	53.1 (136)	
One	46.9 (120)	
**No IPTp-SP**	9.5 (27)	
**Trimester of 1**^**st **^**SP dose**		
1^st^ trimester	0.8 (2)	
2^nd^ trimester	59.8 (150)	
3^rd^ trimester	39.4 (99)	

GA = Gestational age (weeks).

**Table 3:** Relationship between ANC visits, individual factors and use of IPTp

**Factor**	**≤ 3visits**		**≥ 4visits**		*Significance level (χ ^**2**^**; p-value)**	^**#**^**OR (95% CI)**	^**†**^**OR (95% CI)**	**P-value**
	**% (n)**	**95% CI**	**% (n)**	**95% CI**				
**Age (years)**					**7.670; 0.022**			
≤20	40.7 (35)	30.9 – 51.3	25.8 (51)	20.2 – 32.3		**2.0 (1.2 – 3.4)**	2.0 (1.0 – 4.4)	0.069
21-25	34.9 (30)	25.7 – 45.4	36.4 (72)	30.0 – 43.2		0.9 (0.6 – 1.2)	1.5 (0.7– 3.2)	0.254
>25	24.4 (21)	16.6 – 34.5	37.9 (75)	31.4 – 44.8		REF		
**Parity**					0.765;0.682			
Primiparous	34.1 (30)	25.0 – 44.4	29.2 (58)	23.3 – 35.8		1.3 (0.7 – 2.2)	1.2 (0.6 – 2.6)	0.671
Secundiparous	28.4 (25)	20.0 – 38.6	29.2 (58)	23.3 – 35.8		1.0 (0.6 – 1.7)	1.4 (0.7 – 2.8)	0.327
Multiparous	37.5 (33)	28.1 – 47.9	41.7 (83)	35.1 – 48.7		REF		
**Marital status**					**7.693; 0.006**			
Unmarried	35.6 (31)	26.4 – 46.1	20.2 (40)	15.2 – 26.3		**2.2 (1.3 – 3.8)**	**2.5 (1.3 – 4.6)**	**0.006**
Married	64.4 (56)	53.9 –73.6	79.8 (158)	73.7 – 84.8		REF		
**Educational level**					3.740; 0.291			
None	3.8 (3)	1.2 –10.5	7.6 (14)	4.6 – 12.3		0.5 (0.1 – 1.7)	0.8 (0.1 – 5.1)	0.827
Primary	70.0 (56)	59.2 – 78.9	58.9 (109)	51.7 – 65.8		1.6 (0.9 – 2.9)	1.4 (0.4 – 5.5)	0.594
Secondary	21.3 (17)	13.7 – 31.4	29.2 (54)	23.1 – 36.1		0.7 (0.4 – 1.2)	0.8 (0.2 – 3.3)	0.780
Tertiary	5.0 (4)	2.0 – 12.0	4.3 (8)	2.2 – 8.3		REF		
**Fever history**								
Yes	32.5 (26)	23.2 – 43.4	48.4 (93)	41.5 – 55.5	**5.829; 0.016**	**0.5 (0.3 – 0.9)**	**0.5 (0.3 – 0.8)**	**0.009**
No	67.5 (54)	56.6 – 76.8	51.6 (99)	44.5 – 58.5		REF		
**IPTp-SP dosage**								
one	70.3 (45)	58.2 – 80.1	39.1 (75)	32.4 – 46.1	**18.824; 0.001**	**3.7 (2.0 – 6.8)**	NA	
Two or more	29.7 (19)	19.9 – 41.8	60.9 (117)	53.9 – 67.6				

*Determined by Chi-square (Fischer exact) Test; ^**#**^determined from Confidence interval calculator, ^**†**^determined by multinominal logistic regression, OR = Odd ratio, CI = Confidence interval, Values in bold indicate significant associations, Significance level = P < 0.05.

To further investigate the timeliness of ANC attendance, a categorization of ‘early first attendance’ was defined as a first visit to ANC at or before 4 months gestation. Of all respondents only 18.7(50/268) had attended ANC at or before four months of gestation. Women with a fever history were likely (60.0% vs 40.0%; *χ*^2^ = 5.841; P = 0.016) to make an early first ANC visit than those without fever history. No other determinants of early attendance were identified.

### IPTp uptake at ANC clinic

**Figure 1:**
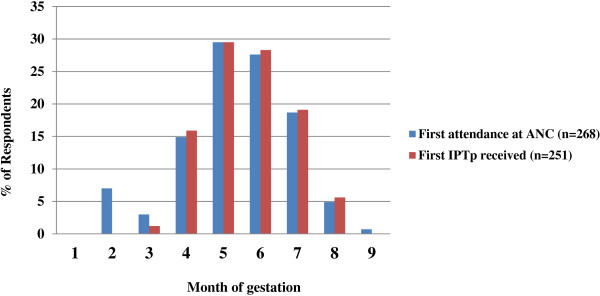
Comparison of timing of first attendance at antenatal clinic with timing of first dose of IPTp in the Mutengene Medical Centre.

Sulphadoxine-pyremathmine treatment was presumptive and administered at an ANC. Among ANC attendees, 90.5% reported receiving at least one dose of SP during pregnancy. However, only 53.1% (136/256) received two or more doses of IPT-SP at an ANC visit. Among women who received SP at least once, only 59.8% reported taking the first dose within the second trimester (19-22weeks) while 39.4% of women received it during the 3rd trimester (26-30weeks) and rarely (0.8%) during the first trimester (<16 weeks) (Table 2). The timing of first IPTp dose within the study population as shown in Figure 1 demonstrates that when IPTp was given, it was generally first given from fourth month of gestation and majority of the women received SP between the fifth and sixth months. This is consistent with the WHO guidelines for delivery of IPTp, which recommends the delivery of first IPT dose at each scheduled visit after ‘quickening’ (16 weeks) to ensure that a high proportion of women received a minimum of two doses of IPT [3]. Furthermore the timing of first IPT dose correlated significantly (r = 0.919; P < 0.001) with the timing of first attendance at ANC clinic (Figure 1).

**Figure 2:**
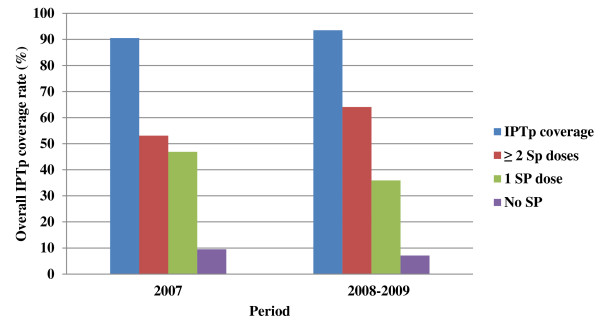
Overall IPTp intake in parturient women in 2007, 2008–2009 in the Mutengene Medical Centre.

The proportion of women receiving the recommended 2 or 3 doses of IPTp-SP increased (p < 0.001) with the number of ANC visits attended (Table 3). Thus women who received one dose (OR = 3.7) were more likely not to have attended four or more visits (Table 3). More so, there was a significant (p = 0.004) association between early first ANC attendance and SP dosage where women who had an early first ANC attendance were more likely (OR = 0.4; 95% CI = 0.2 - 0.7) to receive the recommended two or more SP doses. There was no significant association between age, parity, marital status and IPTp-SP uptake at an ANC. From 2008–2009, a follow-up study showed an increase in IPTp-SP coverage to 93.5% (for women who had received at least one dose) and to 64.1% for those who had received complete IPT (two doses) (Figure 2).

### Association between trimester of first ANC visit, SP dosage and malaria parasite infection status at delivery

A significantly higher (P < 0.001) proportion of women with first ANC visit during the third trimester received only one SP dose during pregnancy compared with those who had two or more doses (Table 4). Equally, the prevalence of microscopic malaria parasitaemia was significantly higher (P = 0.029) among parturient women with first clinic visit during the third trimester when compared with those who enrolled in their second trimester of pregnancy (Table 4). Nonetheless the association between trimester of enrolment and malaria parasitaemia at delivery was only significant among women who had one SP dose. Thus microscopic parasitaemia at delivery was significantly higher (*χ*^2^ = 9.938; P = 0.007) among women who had first ANC attendance in the third trimester and had received one dose (60.9%) or no SP (21.7%) compared to those who had two or more doses (17.4%).

**Table 4:** Association of trimester of first ANC visit with SP dosage and malaria infection status at delivery

**Factor/Trimester**	**First % (n)**	**Second % (n)**	**Third % (n)**	*Significance level
**SP dosage**				
Two or more	33.3 (2)	63.5 (101)	28 (28)	***χ***^**2**^ **= 35.361; p <0.001**
One	50 (3)	34.6 (55)	62 (62)	
No SP	16.7 (1)	1.9 (3)	10 (10)	
**Malaria infection status**				
Microscopic^&^	0 (0)	18.8 (30)	22.5 (23)	***χ***^**2**^ **= 10.795; p = 0.029**
Submicroscopic^#^	66.7 (4)	17.5 (28)	15.7 (16)	
Negative	33.3 (2)	63.8 (102)	61.8 (63)	

*Determined by Pearson Chi-square Test; Significance level = P < 0.05.

^&^Defined as malaria parasitaemia detected by blood smear microscopy and placental histology.

^**#**^Defined as malaria parasitaemia detected by placental histology only.

## Discussion

This study assessed the determinants of ANC clinic attendance and IPTp-SP uptake among parturient women who seek delivery services at the Mutengene Medical Centre, Cameroon to identify facilitating factors for scaling up IPTp-SP coverage/dosage in the study area. Also the study examined the association between time of first ANC clinic attendance, IPTp-SP dosage and malaria parasitaemia at delivery. The study shows a high coverage of ANC attendance in the Mount Cameroon Area, which ranged from one to twelve visits with a median of five visits. The WHO recommendation of four ANC visits was met by more than 70% of the interviewed women. These findings indicate a high rate of antenatal care utilization in the study area, similar to that observed in Gabon [17] and Tanzania [21] and offers the potential for implementing the nationally recommended approaches to the prevention and control of malaria [9]. This high attendance can be explained by the fact that Mutengene Medical Centre is the only public health facility that offers antenatal care, preventive, curative and delivery services at affordable costs when compared with the private medical centres in the health area. In Cameroon, in several health centres and public hospitals, health care services have been made affordable for the middle and low-income population since 2007. Intermittent preventive treatment with SP is freely provided to the mothers and is most often available. Evidence from elsewhere has shown that access to services and costs are serious barriers to utilization of ANC services among the underprivileged and thus making such services affordable to the poor is a necessity [22,23]. Although the timing of ANC was also found to be correct with a large proportion of women having their first visit during the second trimester, more than one third (38%) of women were found to start ANC clinic during the third trimester. Similar findings have been observed in other African countries [24]. A majority of women attended ANC at five months gestation or later and thus represents an opportunity for timely delivery of IPTp-SP. More so, the study showed that the timing of ANC attendance had a significant effect on the uptake of first SP dose. The first SP dose was usually administered after first ANC consultation. In contrast, a previous study in Northeast Tanzania observed that earlier ANC attendance (<20 weeks) had only a limited effect on the uptake of IPTp [25]. Late attendance for some pregnant women (5%) occurred at or after 32 weeks thus leaving insufficient time for the uptake of a second dose of SP before giving birth. This is in line with recent findings in Benin by d’Almeida *et al.*[26].

Factors affecting ANC clinic attendance have been reviewed [11]. Women with a fever history were more likely to make an earlier ANC initiation or more visits (≥4 visits) in this study suggesting that enrolment and attendance are delayed (or the opposite promoted) depending on the health status of the pregnant woman. A study in Tanzania found that having a history of a previous reproductive loss was a strong predictor for an earlier ANC initiation. Secondly marital status was found to be an independent factor associated with frequent ANC clinic visits rather than the timing of the first visit. Similarly, not being supported by the husband or partner was identified as a factor associated with a later antenatal care enrolment in South-eastern Tanzania [21]. On the contrary, a study in rural Uganda found no significant association between marital status and ANC utilization [12]. Findings by Schatz [27] in Malawi have shown that marriage is an economic necessity for women while studies in Kenya [28] and Ghana [29] noted that husbands have an important economic role that influences women’s access to care. More so, some men perceive it as the husband’s responsibility to maintain the well-being of his pregnant wife and to ensure that she attends the ANC. Studies have also shown that adolescent pregnant girls are less likely to attend an ANC and seek timely care for malaria [29,30]. There was no significant relationship between age and ANC attendance after adjusting for covariates in exploratory analysis. Other authors have reported no association [21,31].

In 2007, about 90% of women received at least one dose but only 53% received the recommended two dose SP regimen. This observed coverage is in range with findings from other settings. A cross-sectional study in Gabon reported 84.1% receiving one dose of SP and 57.4% received at least two doses [17]. In Burkina Faso, 93.5% took any dose of IPTp/SP and two or more doses of SP were taken by 57.6% of the women [32]. In 2008–2009, an increase in the coverage rate of IPT-two doses to 64% was recorded probably due to continuous awareness campaigns organized throughout the country. Similarly, in Benin, the rate of IPT-SP coverage for those who had received complete IPT increased to 68.4% in 2009 [26]. In the study area, the delivery of the first IPTp-SP dose was timely, generally between 16-24weeks, which is in accordance with the WHO implementation guideline [3]. Firstly, the low coverage of the second SP dose compared to that of first dose in study area could be due to fewer ANC visits (≤3visits). Women who had fewer ANC visits were almost four times likely not to take the recommended SP dosage. This finding confirms reports from Benin and Cameroon where IPT administration varied with the number of prenatal consultations [26,33]. Comparable to findings by Bouyou-Akotet *et al*. [17] in Gabon, it is noteworthy that 39% of parturient women with more than three ANC visits had partial SP uptake which is a evident that several ANC visits does not necessary ensure complete IPTp uptake, thus identification of facilitating factors to enhance uptake of complete IPTp dose warrants investigation. Secondly, achievement of full SP dose was also prevented by late enrolment at the ANC, frequently delayed until the third trimester and at or after 32 weeks of gestation for certain women, in line with reports from Benin [26]. This study further shows that women who attended first ANC clinic in the third trimester and had one or no SP dose frequently had microscopic parasitaemia at delivery suggesting that late ANC may have important implications in the prevalence of malaria parasitaemia in women at delivery.

## Conclusion

Marital status and fever history independently influenced ANC clinic attendance while uptake of one SP dose was associated with late first ANC visit and fewer clinic visits. Thus earlier ANC clinic attendance may improve coverage of two or more SP doses in the study area. Given that about 40% of parturient women who had recommended number ANC visits (≥4visits) had partial SP uptake suggests that other factors besides number and timing of ANC clinic visits affect the delivery, access, and use of interventions to prevent malaria in pregnancy in the study area. Thus identification of barriers to the provision and uptake of IPTp warrants further investigation. More so, educating pregnant women on focused ANC clinic visits is recommended.

## Competing interests

The authors declare that they have no competing interests.

## Authors’ contributions

JKAK: Conception and design of study, data collection, analysis, interpretation and manuscript preparation. EAA: Supervision and critically reading of manuscript for important and intellectual content. JKAK TOA, RNM, HFC, RBT: Data collection on IPTp during the 2008–2009 survey. NB and JMNM: Contributed in placental histopathological studies. ESE: Design of study and critically reading of manuscript for important and intellectual content; MTB: Supervision and revision of manuscript. All authors read and approved the manuscript.
